# Patch-Wise Deep Learning Method for Intracranial Stenosis and Aneurysm Detection-the Tromsø Study

**DOI:** 10.1007/s12021-024-09697-z

**Published:** 2025-01-15

**Authors:** Luca Bernecker, Ellisiv B. Mathiesen, Tor Ingebrigtsen, Jørgen Isaksen, Liv-Hege Johnsen, Torgil Riise Vangberg

**Affiliations:** 1 Department of Clinical Medicine, UiT the Arctic University of Norway, Tromsø, Norway; 2 PET Imaging Center, University Hospital North Norway, Tromsø, Norway; 3 Department of Radiology, University Hospital North Norway, Tromsø, Norway; 4 Department of Neurology, University Hospital North Norway, Tromsø, Norway; 5 Department of Neurosurgery, Ophthalmology, and Otorhinolaryngology, University Hospital of North Norway, Tromsø, Norway

**Keywords:** Intracranial Stenosis, Aneurysms, Deep learning, Detection algorithm

## Abstract

Intracranial atherosclerotic stenosis (ICAS) and intracranial aneurysms are prevalent conditions in the cerebrovascular system. ICAS causes a narrowing of the arterial lumen, thereby restricting blood flow, while aneurysms involve the ballooning of blood vessels. Both conditions can lead to severe outcomes, such as stroke or vessel rupture, which can be fatal. Early detection is crucial for effective intervention. In this study, we introduced a method that combines classical computer vision techniques with deep learning to detect intracranial aneurysms and ICAS in time-of-flight magnetic resonance angiography images. The process began with skull-stripping, followed by an affine transformation to align the images to a common atlas space. We then focused on the region of interest, including the circle of Willis, by cropping the relevant area. A segmentation algorithm was used to isolate the arteries, after which a patch-wise residual neural network was applied across the image. A voting mechanism was then employed to identify the presence of atrophies. Our method achieved accuracies of 76.5% for aneurysms and 82.4% for ICAS. Notably, when occlusions were not considered, the accuracy for ICAS detection improved to 85.7%. While the algorithm performed well for localized pathological findings, it was less effective at detecting occlusions, which involved long-range dependencies in the MRIs. This limitation was due to the architectural design of the patch-wise deep learning approach. Regardless, this can, in the future, be mitigated in a multi-scale patch-wise algorithm.

## Introduction

Intracranial atherosclerotic stenosis (ICAS) and intracranial aneurysms are two common diseases in middle-aged and older people. ICAS refers to a narrowing of the arterial lumen, in this study we consider only narrowing of 50% or more. This narrowing is typically caused by atherosclerosis, a condition where fatty deposits build up on the inner walls of the arteries, having hemodynamic consequences. An intracranial aneurysm is caused by a weakened area in the wall of an artery that, over time, expands and results in a localized outpouching and exposed to hemodynamic stress.

It is estimated that ICAS cause 5–10% of ischemic strokes (White et al., 2005; Holmstedt et al., 2013; Turan et al., 2010), and approximately 14% of patients with symptomatic ICAS experience recurrent stroke events within three years (Hurford et al. 2020). Stroke ranks as the second most common cause of death worldwide, imposing significant economic strain due to the costs associated with treatment and post-stroke care (Johnson et al. 2019). Aneurysms may rupture, causing aneurysmal subarachnoid hemorrhage with an incidence rate of 5.7 per 100.000 a year (95% CI 5.4–6.0) in Norway (Iversen 2022; Øie et al. 2020). Despite advancements in both medical and surgical interventions, the rupture of an aneurysm continues to be associated with considerable rates of case fatality and severe disability (Iversen 2022).

Time-of-flight magnetic resonance angiography (TOF-MRA) is a noninvasive method for identifying ICAS and aneurysms that do not involve harmful radiation or contrast agents and can offer greater detail than other image modalities (Choi et al. 2007; Isoda et al. 2000). There is considerable interest in creating automated methods for detecting ICAS and aneurysms from TOF-MRA to aid in the clinical evaluation of these pathologies as they often are discovered incidentally, aneurysms in particular, and because the increased use of medical imaging places a greater workload on radiologists (McDonald et al. 2015).

Several different deep-learning models have been introduced for ICAS and aneurysm detection in TOF-MRA. For aneurysms, one of the best-performing algorithms, which is based on a patch-wise structure, has a sensitivity of 87.1% (Joo et al. 2020). For TOF-MRA ICAS detection, a 2D Yolo-based detection algorithm was introduced to achieve a sensitivity of 64.2% with a positive predictive value of 83.7% (Qiu et al. 2022). While the results for intracranial aneurysms are clinically acceptable, a good solution for TOF-MRA-based intracranial stenosis detection is lacking. The aim of this paper was to develop a general algorithm for detecting intracranial aneurysms and ICAS on TOF-MRA by further refinement of a successful patch-wise algorithm (Joo et al. 2020) and to evaluate the model’s performance with respect to sensitivity and specificity, but also the positive predictive value which considers the disease’s prevalence.

## Materials and Method

The study was approved by the Regional Committee of Medical and Health Research Ethics Northern Norway (REK-Nord 619939) and carried out in accordance with guidelines at UiT The Arctic University of Norway. All participants gave written informed consent before participating in the study.

### Dataset

The seventh Tromsø study is a comprehensive population health study in the municipal city of Tromsø, Norway, encompassing various health-related aspects. In a sub-study, cerebral magnetic resonance imaging (MRI) was collected in 1878 participants comprising 53.2% women with a mean age of 63 years and 46.2% men with a mean age of 64 years. Participants underwent imaging at the University Hospital of North Norway, Tromsø, utilizing a 3 Tesla (3T) Siemens Skyra MR scanner (Siemens Healthcare, Erlangen, Germany). Only the time-of-flight magnetic resonance angiography (TOF-MRA) images were used in this study. The TOF-MRA sequence was a 3D transversal fast low-angle shot sequence with flow compensation. Key acquisition parameters: repetition time/echo time 21/3.43 ms, parallel imaging acceleration factor 3, field of view of 200 × 181 mm, slice thickness 0.5 mm, 7 slabs with 40 slices each, and reconstructed image resolution 0.3 × 0.3 × 0.5 mm.

There were 1847 participants evaluated for ICAS (Johnsen et al. 2023). The degree of stenosis for the ICAS was graded using the Warfarin-Aspirin Symptomatic Intracranial Disease (WASID) (Chimowitz et al. 2005) and the visual grading system for intracranial arterial stenosis on TOF-MRA (MRA-VICAST) (You et al. 2022) methods. In case of disagreement between the methods, a consensus was reached between two neuroradiologists. ICAS was characterized as a focal narrowing of the intracranial arterial flow diameter of at least 50% in the WASID method (Samuels et al. 2000). The severity of stenosis was classified into three groups:50–69% (moderate), 70–99% (severe), and 100% (occlusion) (Johnsen et al. 2023). For further details on the ICAS rating, see Johnsen and coworkers (Johnsen et al. 2023). There were 111/1847 (6.0%) participants with ICAS in our dataset. Of these 88/1847 (4.8%) participants had moderate stenosis, 20/1847 (1.1%) severe stenosis, and 3/1847 (0.2%) full occlusions. Several participants had multiple pathological findings resulting in total, of the 111/1847 (5.9%) participants, we counted 126 moderate, 26 severe, and 4 full occlusions.

There were 1862 participants evaluated for saccular intracranial aneurysms (Johnsen et al. 2022). A saccular aneurysm was defined as a focal saccular dilation that was greater or equal to 2 mm in size on an intracranial artery. There were 122/1862 (6.6%) participants with a total of 131 aneurysms in the sample. Most aneurysms were less than 5 mm, with 65/1862 (3.5%) between 2 and 2.9 mm, 48/1862 (2.6%) between 3 and 4.9 mm, 18/1862 (1.0%) between 5 and 6.9 mm, and 9/1862 (0.5%) ≥ 7 mm.

### Processing of TOF Images

#### Circle of Willis Extraction and Moving to MNI Space

We skull-stripped the TOF images with SynthStrip (Hoopes et al. 2022) and then affine-transformed the image into Montreal Neurological Institute (MNI) space with the Advanced Normalization Tools in Python (ANTSpy) version 0.5.1 (github.com/ANTsX/ANTsPy) with a TOF-MNI template as target (Mouches and Forkert 2019). This transformation reduced the resolution from 0.3 × 0.3 × 0.5 mm to 0.5 × 0.5 × 0.5 mm. A region of interest (ROI) was constructed in MNI space (102 × 73 × 78 mm cube centered in the pituitary gland, MNI coordinates 0,3,-33 mm) covering the circle of Willis, the internal carotid, basilar, and vertebral arteries down to the base of the skull, and the largest arteries branching off from the circle of Willis, the anterior, middle and posterior cerebral arteries. This region included all annotated aneurysms and ICAS from the respective dataset. These cropped TOF images in MNI space were used in the subsequent analysis.

#### Arterial Segmentation

**Fig. 1:**
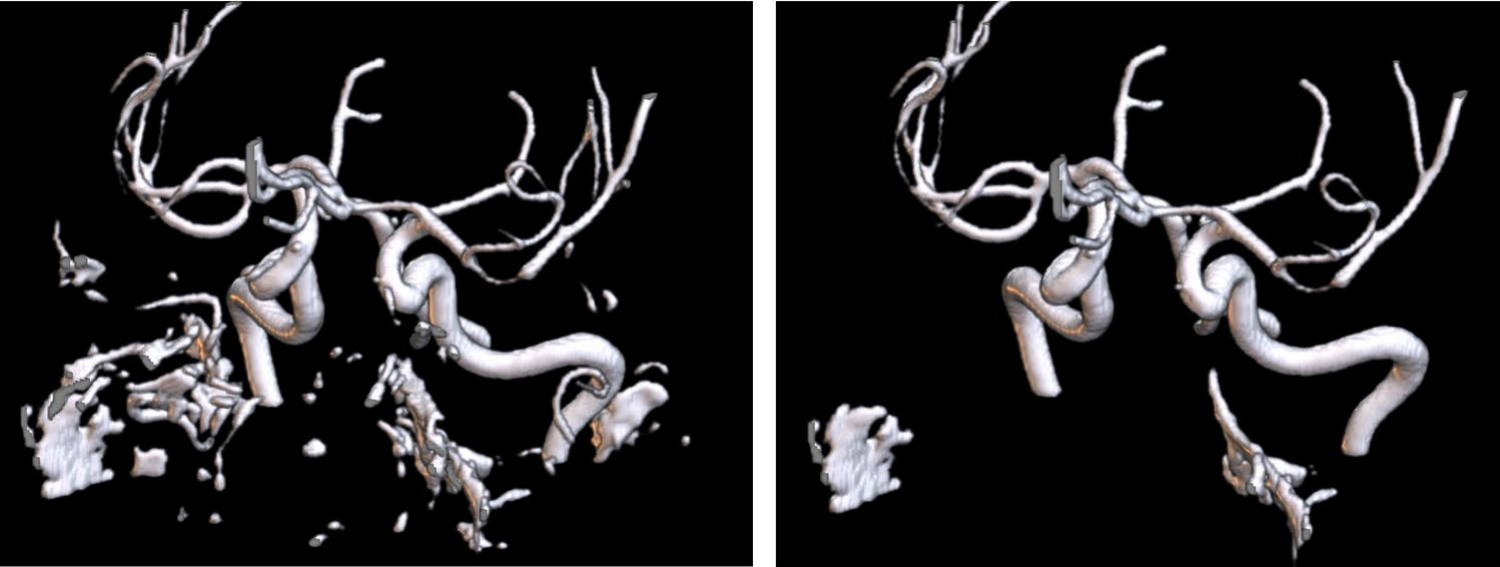
Example of vessel segmentation with the FFCM algorithm. On the left is the TOF image after the FFCM segmentation. On the right is the result after removing clusters of less than 177 ml

The arteries in the cropped TOF images in MNI space were segmented with the Fast Fuzzy c-Means (FFCM) algorithm (Cui et al. 2024). After the segmentation, we removed small clusters that were not arteries by removing isolated clusters of 175 ml (3500 voxels) or less. Figure 1 illustrates the effect of the FFCM segmentation and subsequent removal of small clusters.

### Data Preparation for Deep Learning and Augmentation

The data was split into 80/5/15, instead of the usual 75/10/15 ratio to compensate for little training data. For the patch-wise deep learning algorithm, we augmented the 16 × 16 × 16 mm (32 × 32 × 32 voxels) patches by flips along the three axes and allowing an offset of 4 mm of the ICAS or aneurysm, which was initially centered in the middle of the patches. For the patches without pathological findings, we used the same coordinates in the MNI space as for pathological findings but extracted the patches from 400 healthy participants randomly sampled from the full set of healthy participants.

The false positives were evaluated after an initial trial, which revealed that there were too many false positives in the internal carotid artery (ICA) and the bifurcations between ICAs and the middle cerebral arteries for the aneurysm classification. Therefore, an oversampling approach was deployed to allow the algorithm to learn the normal representation of these regions by sampling these regions from the participants without any pathology. This resulted in 90 aneurysms with an augmentation to 237 with a binary classification ratio of patches of 4:1, where 1 stands for the aneurysm cases.

The same process was done for ICAS, where the false positive rates in the posterior cerebral arteries and the branches extending from the left and right cerebral arteries were extracted from healthy participants. This resulted in 83 ICAS augmented to 255 with a ratio of 5:1, where 1 represents the ICAS. However, during the validation and testing, the ratio is kept at 1:1.

### Deep Learning Patch-Wise Classification

**Fig. 2:**
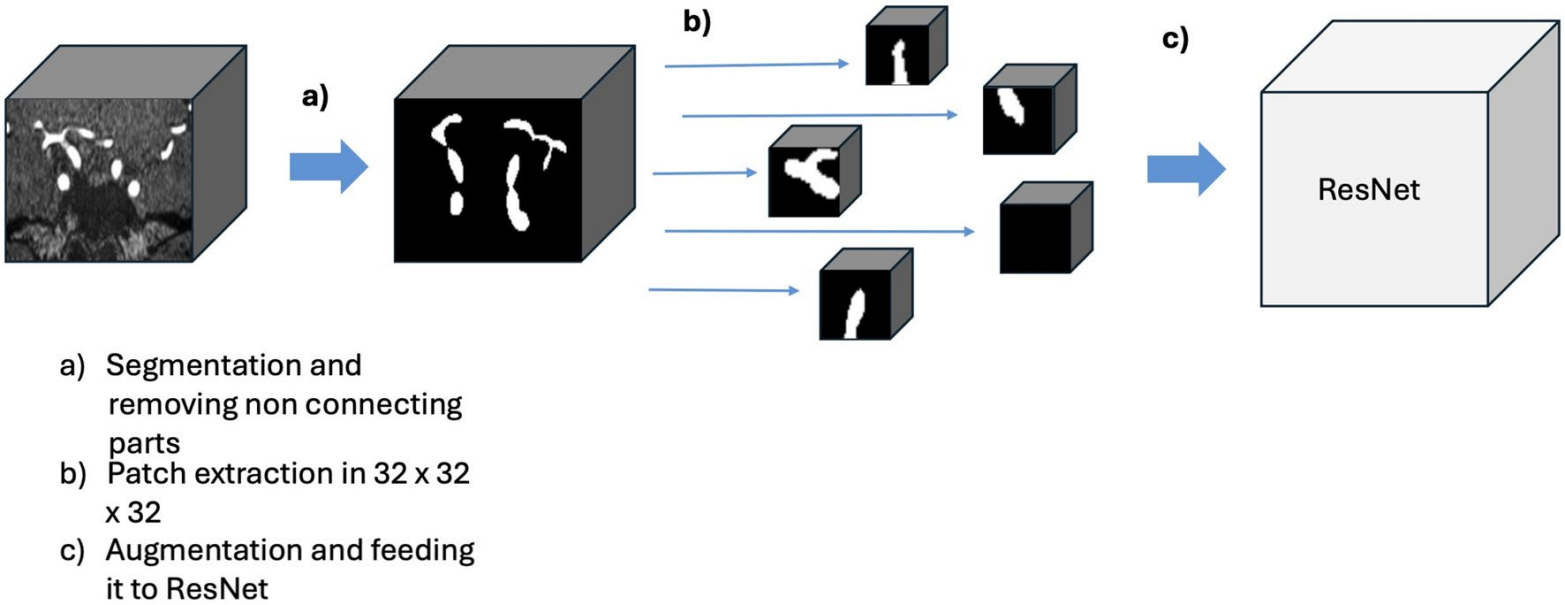
Algorithm to train the deep learning model. Once the circle of Willis region is extracted, we applied the segmentation in (**a**) and removed further non-connecting parts. Then we split it into patches in (**b**) and in (**c**) we augmented the data and trained the deep learning model

We applied a 3D ResNet50 (Szegedy 2017) to the 32 × 32 × 32 patches for 50 epochs with the Adams optimization algorithm, with an initial learning rate of 0.0005 and an early stop based on the validation set (Kingma et al. 2014). This can be seen in Fig. 2, which shows steps (a) where the segmentation was applied, (b) the extraction patches, and (c) the augmentation of the patches to train the ResNet50 model. This procedure was followed for ICAS, while for the aneurysms step, a) was skipped due to the lower accuracy of the FFCM algorithm in segmenting vessels with aneurysms.

#### Voting Algorithm

**Fig. 3:**
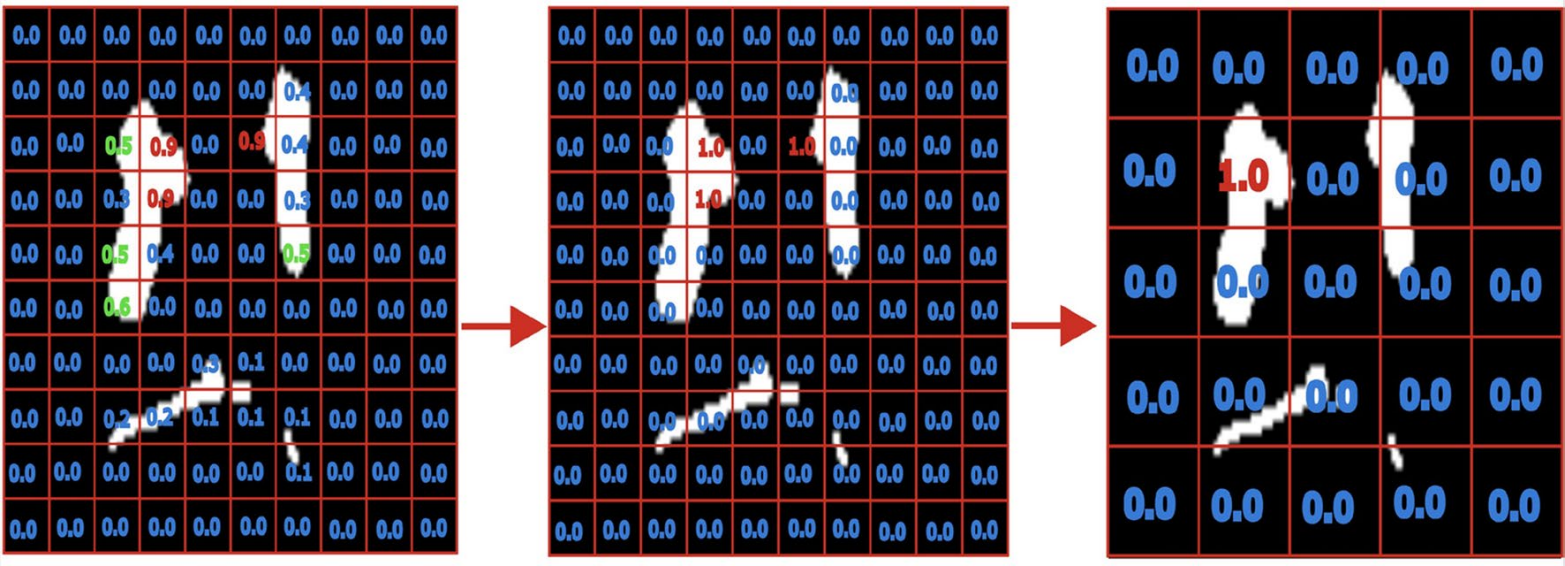
On the left, we first see part of the voting algorithm, evaluating all images in patches. The second part is to threshold them to 0 or 1. The last is to connect and only use large, connected parts of rated ones

After training the ResNet50, we performed inference on the ROI, which was cropped by the preprocessing with a stride of 4 and constructed a 3D representation of those results. This can be seen as a convolution window of shape 32 × 32 × 32 sliding with stride 4 through the image. The overlapping windows resulted in 40,608 classifications, which spans the probability space of 40,608 voxels. Then, all voxels resulting from the patch classification were thresholded by 0.99954 and finally binarized, as seen in the first step in Fig. 3. The figure is a simplified version, where we do only portray the adjacent predictions in 2D, while in the detailed algorithm the classifications overlap. In the second step we count the adjacent positive predictions. If there are 25 adjacent positive predictions, we consider this location as a pathological finding.

## Results

### Aneurysm Detection

**Fig. 4:**
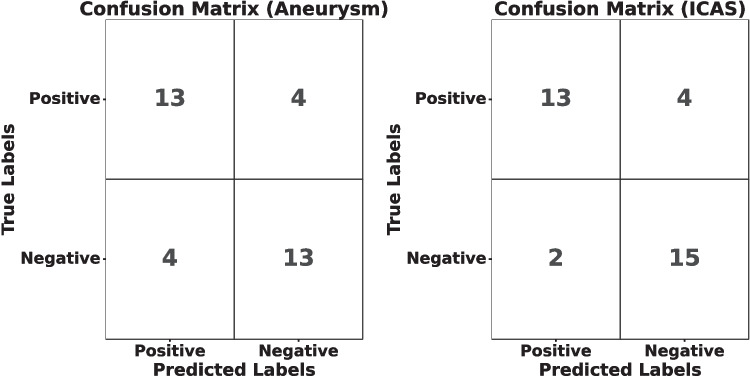
Confusion matrices for both aneurysms and intracranial atherosclerosis stenosis (ICAS) for the testing sets

The 3D ResNet50 patch-wise classification achieved an accuracy of 96.3% on the training set and a validation accuracy of 87.0% for detecting intracranial aneurysms. For the participants’ test set, the overall specificity and sensitivity were 76.5% each, with the detailed distribution between true positive, false positive, true negative and false negative seen in Fig. 4.

The performance metrics for participants are reported in Table 1, where we also report the sensitivity for aneurysms less than 4 mm and greater than 4 mm to assess the algorithm’s sensitivity to aneurysm size since the risk of rupture increases with the size of the aneurysm (Wiebers 2003; Bijlenga et al. 2013). Table1shows that the algorithm had a sensitivity for detecting aneurysms below 4 mm of 70.0% and above 4 mm of 85.7%. The average size of the false negative aneurysms was 3.57 (SD ± 1.15). The aneurysms in the posterior cerebral artery, combined with the middle cerebral arteries, had a sensitivity of 88.9%, while for the ICA and anterior communicating artery (Acom) combined, the accuracy was 57.0%. Accounting for the prevalence in the population sample of 6.6%, the positive and negative predictive values (Guggenmoos-Holzmann and van Houwelingen 2000) were 17.7% and 98.0%. The results are further illustrated with a confusion matrix Fig. 4 presenting the number of correct and incorrect predictions per class.

**Table 1:** Performance of intracranial aneurysm detection on the test set

Characteristics	Test set
Total number of Aneurysms	17
Average Size	4.57 (± 2.52)
Accuracy	76.5% (26/34)
PPV	76.5% (13/17)
Sensitivity	
* >* 4 mm	70.0% (7/10)
* <* 4 mm	85.7% (6/7)
Specificity	76.5% (13/17)
Prev-PPV^*1*^	17.7%
Prev-NPV^*1*^	98.0%
*Sensitivity based on location*	
Internal carotid artery	60.0% (3/5)
Middle cerebral artery	80.0% (4/5)
Posterior cerebral artery	100.0% (4/4)
Acom	50.0%(1/2)
Basilar	100.0% (1/1)

^*1*^Predictive values including prevalence according to (Guggenmoos-Holzmann and van Houwelingen 2000)

### Stenosis Detection

The patch-wise classification for ICAS achieved an accuracy of 88.72% and 87.69% during training and validation, respectively. In the test set, the algorithm had an 82.4% accuracy on the participant level, with a sensitivity of 76.5% and specificity of 86.7%, which is further detailed in a confusion matrix in Fig. 4 and reported in Table 2. Diagnostic performance based on location showed that detecting stenosis in the ICA had the greatest sensitivity of 88.9%, whereas, in the middle and posterior cerebral arteries, the sensitivities were 66.7% and 75.0%. There was only one stenosis in the anterior cerebral artery, which was missed by the algorithm, giving a sensitivity of 0.0%. The positive predictive (PPV) value was 88.2% on the balanced dataset. Accounting for the population prevalence of 6.0% for ICAS, the PPV and NPV values were 27.2 and 98.3%. The model’s sensitivity for moderate and severe stenosis was 85.7% (12/14), while the sensitivity for occlusions was 33.3%, although the small number of severe stenosis and occlusion cases places some uncertainty on the sensitivity estimates.

**Table 2:** Performance of intracranial stenosis detection characteristics on the test set

Characteristics	Test set
Total number of ICAS	17
Accuracy	82.4% (28/34)
PPV	86.7% (13/15)
Sensitivity *Moderate*	91.2% (11/12)
*Severe*	50.0% (1/2)
*Occlusion*	33.3% (1/3)
Specificity	88.2% (15/17)
Prev-PPV^*1*^	27.2%
Prev-NPV^*1*^	98.3%
*Sensitivity according to location*	
Internal carotid artery	88.9% (8/9)
Middle cerebral artery	66.7% (2/3)
Posterior cerebral artery	75.0% (3/4)
Anterior cerebral artery	0.0% (0/1)

^*1*^Predictive values including prevalence (Guggenmoos-Holzmann and van Houwelingen 2000)

## Discussion

Based on patch-wise classification and a relatively small dataset, our deep learning methods achieved a participant-wise accuracy of 76.5% for intracranial aneurysms and 82.4% for ICAS. To our knowledge, the accuracy for ICAS is the highest reported to date. Although the overall accuracy for detecting aneurysms was less than that of other recent studies, the algorithm performed better for the larger and more clinically relevant aneurysms (79.2% accuracy for aneurysms *>* 4 mm). The false positives were mainly in the cavernous segment of the ICA. In sum, the results demonstrate the potential of ResNet patch-based architectures for detecting cerebrovascular pathology on TOF-MRA.

There has been extensive research into deep-learning algorithms for detecting intracranial aneurysms on TOF-MRA with algorithms based on convolutional neural network (CNN) (Nakao et al., 2018), U-Net (Claux et al. 2023; Lehnen et al., 2022), and ResNet (Joo et al. 2020; Ueda et al. 2019; Li et al., 2024). The best-performing algorithms are similarly based on ResNet architectures like ours, with sensitivity between 86 and 92% and specificity between 92 and 98% (Joo et al. 2020; Li et al., 2024). The lower accuracy of our aneurysm detection algorithm may be due to the relatively small training dataset. The high-performing model from the 2020 study by Joo and coworkers (Joo et al. 2020) was trained on a large dataset comprising 468 aneurysm cases, whereas our model was trained on 92 scans with aneurysms. Despite the similarities in algorithms, the substantial difference in the size of the training data is a crucial factor influencing model performance. Furthermore, this study is based on population data, which differs from patient samples. For a clinically relevant algorithm, it would benefit from including ruptured and giant aneurysms. Direct comparison of algorithmic performance without considering the size and nature of the training set is difficult, and our results are comparable to studies using similarly sized datasets.

Compared to intracranial aneurysm detection, there has, to our knowledge, only been two previous papers on detecting ICAS on TOF-MRA despite the comparable severity and prevalence of the two diseases. The first paper on ICAS detection on TOF-MRA using deep-learning methods was designed to detect ICAS only in the ICA (Chung et al. 2020). Their approach involved semi-manually extracting the ICA and subsequently employing a 64 × 64 × 64 mm patch-wise 3D SE-ResNet (Hu 2018) for stenosis detection, achieving an 81.0% accuracy for detecting stenosis in the ICA. Another study using the YOLOv5 model trained on 291 stenoses achieved a sensitivity of 62.4% and a positive predictive value of 83.7% (Qiu et al. 2022). Like us, the authors noted that the highest sensitivity was for detecting stenosis in the ICA (83.7%).

Several implementation details were essential for achieving an accuracy of over 80% for the ICAS detection algorithm. The vessel segmentation step greatly improved model performance. Also, patches from the false positive regions were added to the training data, changing the training ratio from 4:1 to 5:1. This modification increased the specificity from 76.4 to 86.2% while maintaining the same sensitivity. Moderate to severe stenosis has serious implications for stroke, where our model had an accuracy of 87%. Nonetheless, for occlusions, which are the most critical form of ICAS, our model performed poorly. This is likely because patch-wise deep-learning architectures are not capable of describing long-range dependencies. This limitation of the method could be overcome by multiscale techniques (Banik et al. 2020).

**Fig. 5:**
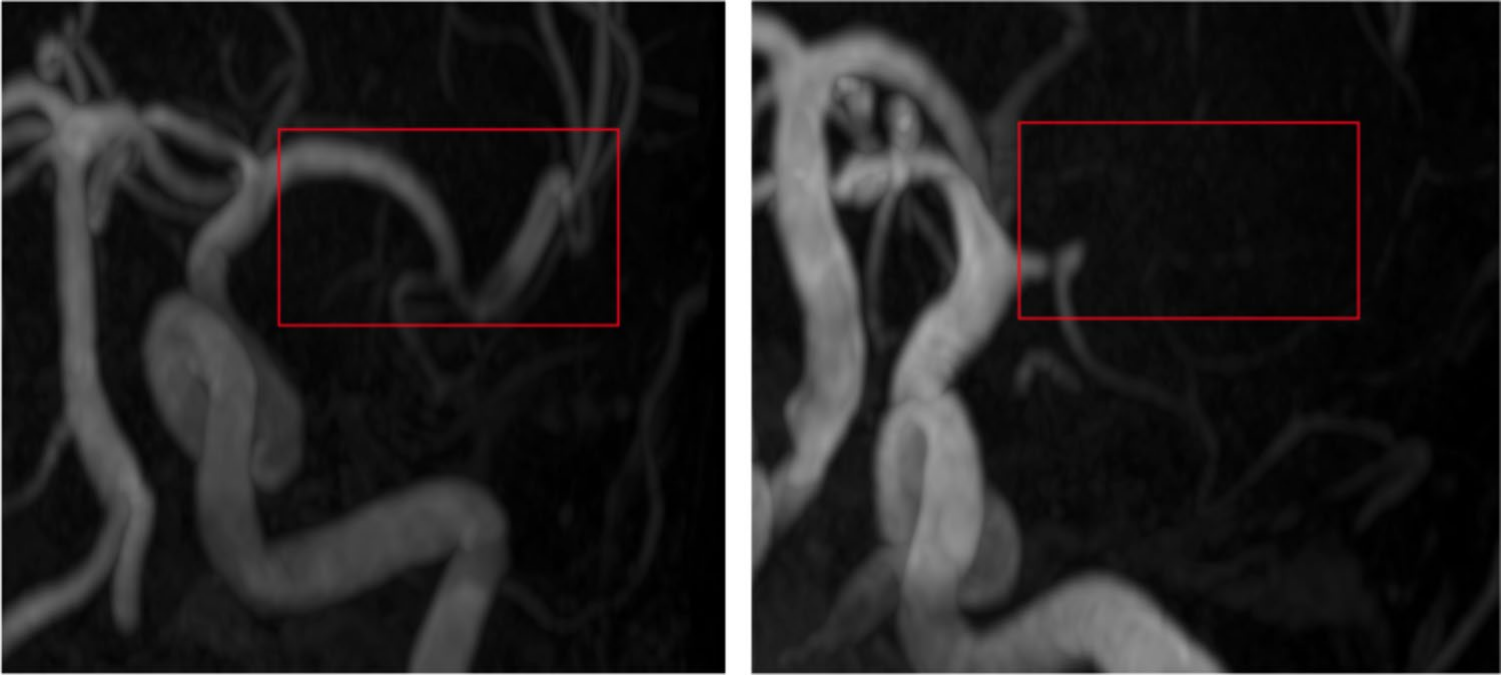
To the left is a normal middle intracranial artery with regular blood flow (red box), and to the right is a false negative with an occlusion, which stopped blood flow, in the anterior intracranial artery

**Fig. 6:**
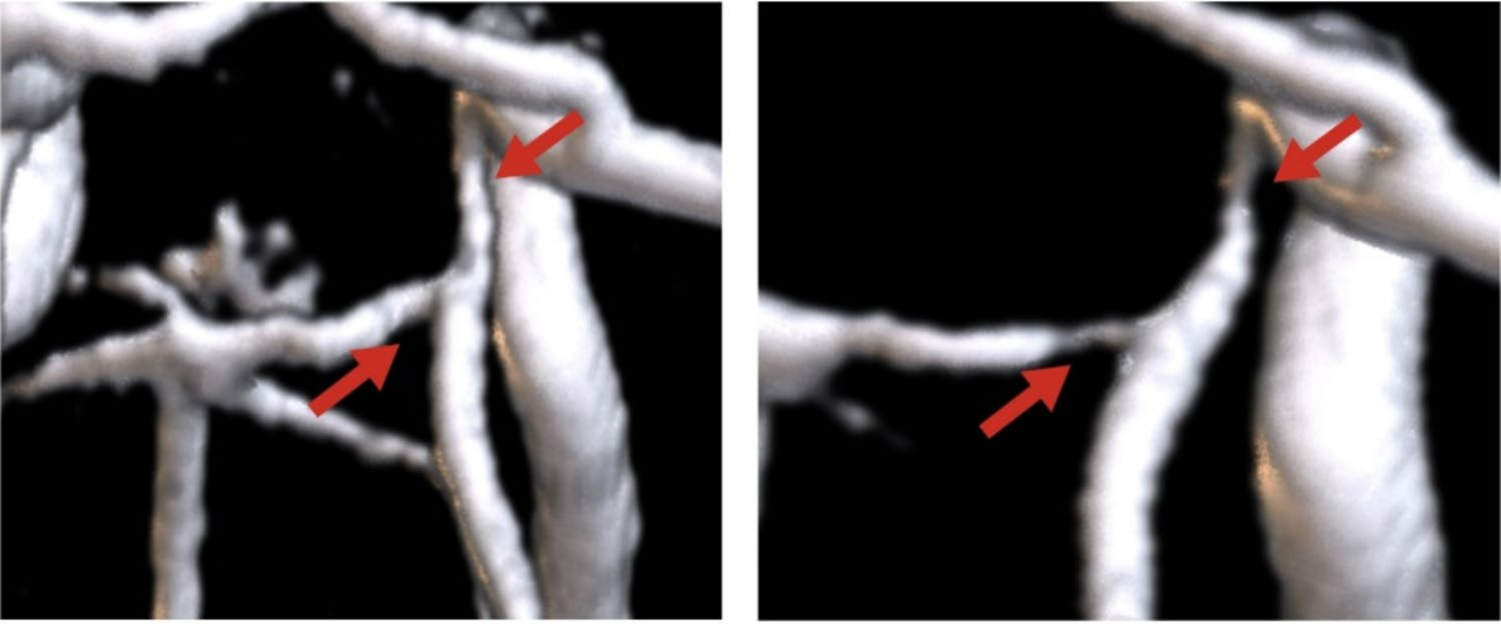
False positive for intracranial stenosis. On the left, we depicted a manually thresholded image, and on the right side, the same image with the FFCM applied. The segmentations error introduces signs of stenosis, while this is not the case, resulting in a false positive

The analysis of false positives in our ICAS detection model revealed primary sources of error. First, as illustrated in Fig. 1, the segmentation algorithm was not optimal, leading to inaccuracies in vessel delineation. Second, our current model is unable to detect occlusions. In cases of occlusion, no thinning of the lumen diameter is observed because the TOF-MRA technique depends on the flow, which results in a different appearance of full occlusions. Instead, there is a missing signal with long-range dependency. The algorithm is primarily trained on moderate stenosis, which is characterized by lumen thinning. Consequently, occlusions and severe stenosis, often appearing as short signal loss or occlusions with extended signal absence during segmentation, present challenges for accurate detection. Such an occlusion can be seen in Fig. 5. On the left we illustrate the TOF-MRA of a healthy participant, while on the right side we can see an occlusion. The lumen illustrate the blood flow, which is extremely decreased on the right side, while clearly visible on the left side. Last, in Fig. 6a false positive for ICAS was detected, which shows that the segmentation falsely segmented the artery too narrowly. To address these limitations and improve the model’s performance, we propose two key strategies. First, developing an enhanced segmentation algorithm that can accurately delineate vascular structures, including vessels with aneurysms or stenosis is crucial. Precise segmentation is prerequisite for reliable stenosis detection and localization. Second, expanding the training dataset with a larger and more diverse set of cases is essential. Increased data diversity can improve the model’s ability to distinguish between stenosis and occlusion, thereby reducing false positive rates.

### Strengths and Limitations

The study’s strengths include a single algorithmic design for detecting intracranial aneurysms and ICAS. Using a relatively small training dataset, we outperformed other current methods for ICAS detection in TOF-MRA (Qiu et al. 2022; Chung et al. 2020). Although our algorithm enables localization and classification, its patch-wise approach limits its ability to detect occlusions where large portions of the arteries are not visible, and the detection is not graded. Additionally, the layering of several computationally intensive algorithms results in high run times and memory requirements for inference. However, there is considerable potential in optimizing the code, for example, by running inferences only on patches with a high probability of containing vessels. There are indications that the dataset we used is too small for proper training of the models, and lastly, we did not validate the algorithms on independent datasets, which would allow a more thorough evaluation of the models.

## Conclusion

In this study, we explored the adaptation of a deep learning algorithm initially designed for intracranial aneurysms (Joo et al. 2020) to detect aneurysms and ICAS on TOF-MRA images. We achieved accuracies of 76.5% and 82.4% for aneurysms and ICAS. The classification could be enhanced further by including demographic and clinical data, which correlate highly to ICAS (Holmstedt et al. 2013). Thus, we believe that with a robust and comprehensive dataset, along with ongoing improvements to the model, it is possible to enhance the ICAS detection further so it achieves a clinically acceptable accuracy.

## Data Availability

The data used in the analysis may be obtained via an application to the Tromsø Study (tromsous@uit.no). Inference code for the deep learning backbone can be found on github (github.com/LucaBernecker/Aneu_Steno_Patch_wise).
